# Microcavity electrodynamics of hybrid surface plasmon polariton modes in high-quality multilayer trench gratings

**DOI:** 10.1038/s41377-018-0009-x

**Published:** 2018-06-22

**Authors:** Xiaoyi Liu, Jinbo Gao, Jinsong Gao, Haigui Yang, Xiaoyi Wang, Tongtong Wang, Zhenfeng Shen, Zhen Liu, Hai Liu, Jian Zhang, Zizheng Li, Yanchao Wang, Qiang Li

**Affiliations:** 1 Key Laboratory of Optical System Advanced Manufacturing Technology, Changchun Institute of Optics, Fine Mechanics and Physics, Chinese Academy of Sciences, Changchun, 130033 China; 2 University of the Chinese Academy of Sciences, Beijing, 100039 China

## Abstract

In common plasmonic structures, absorption and radiation losses are often mutually restricted and can seriously influence the device performance. The current study presents a compound structure composed of multilayer grating stripes and multilayer shallow trenches. A small depth was adopted for the trench configuration to exclude the extra bend loss. These two sections supported Fabry–Perot resonance and cavity modes, respectively, with hybrid modes formed through intercoupling. In addition, the total loss for the entire framework was clearly reduced due to the introduction of the trench geometry, indicating that both absorption and radiation losses were successfully taken into consideration in the compound structure. Significantly, such a low loss realized by the hybridization of surface plasmon polariton modes has rarely been seen before. Moreover, the debatable relationship between the total and partial quality factors was described for the first time based on a hybrid mode analysis to establish a new approach to investigate the different resonance modes. In the detailed calculation process, the relative electric field intensity was first adopted to stipulate the effective areas for the various modes, which is more reasonable than using the common definition that is based on a unit structure. The multilayer trench grating exhibited a relatively low loss without weakening energy localization, which is significant in the design of plasmonic devices.

## Plasmonic gratings: finding quality in the trenches

Losses associated with confining surface waves inside metal–insulator–metal waveguides can be minimized by optimizing trench structures in microscale gratings. Jinsong Gao from the Chinese Academy of Sciences and colleagues used direct laser patterning to create 450-nanometer deep periodic patterns in silicon wafers, then coated the waveguide with five alternate aluminum and silicon layers. In the higher, stripe-shaped portions of the device, multiple modes of trapped surface plasmon polaritons appeared, following a pattern known as Fabry-Pérot resonance. Trenches, however, had surface waves generated by the microcavity shape. By customizing the grating dimensions, the team merged the two types of resonances into new modes with exceptionally low levels of radiation and absorption loss. Analytical treatments revealed quality factors of the hybrid modes were better predicted using relative electric field intensities than periodic cell lengths.

## Introduction

The utilization of surface plasmon polaritons (SPPs) to regulate the optical field and light–matter interaction on a subwavelength scale is a nanophotonics research hotspot^1–5^. As a special surface wave, SPPs can only propagate along the metal–insulator interface within a relatively thin range. In comparison, attenuation will simultaneously execute along the vertical direction. Accordingly, SPPs are capable of confining energy and enhancing local electromagnetic fields, which can lead to potential applications for surface-enhanced spectroscopy^6,7^, spacer^8,9^, solar cells^10,11^, hot carrier extraction technology^12,13^, metamaterials^14,15^, and high-integration optical devices^16,17^, to name a few. To date, a variety of plasmonic microstructures have been extensively investigated, such as optical antennae^18^, grooves^19^, and one- or two-dimensional gratings^20,21^. The SPP penetration depth on the metal side is much smaller than that on the insulator side. As a result, a configuration based on an insulator layer sandwiched between two metal layers can be employed to further constrain light, thereby offering a wide range of applications for plasmonic waveguides or resonators^22,23^.

Although such a metal–insulator–metal (MIM) structure can show remarkable field localization, significant absorption loss is an inevitable problem due to the presence of the metal, which significantly influences surface wave propagation^24^. A relatively low absorption loss and long propagation distance can be obtained by weakening the metal’s binding to the light, although this sacrifices certain characteristics of the MIM configuration^25^. In addition, the radiation loss for an open architecture structure without a closed shell cannot be ignored^24^. The coupling and hybridization between different modes may suppress loss, which is usually observed in localized resonances supported by nano-disks or patches^26–29^. The coupling phenomenon can also be excited through propagating and non-propagating plasmonic modes supported by nano-rods, tubes, or whispering gallery cavities^30–33^. However, research on low-loss structures based on hybrid SPP modes is rarely reported. Very recently, our previous work demonstrated an SPP-mode hybridization phenomenon based on a multilayer trench grating geometry^34^, which led to the realization of a large absorption quality factor (*Q*) in the trench configuration but lacked a quantitative analysis and verification of the entire loss inhibition. Furthermore, the microcavity electrodynamic processes in the hybrid SPP modes, specifically absorption, radiation, and connections between various losses, which are significant to the design of the plasmonic devices, all remain unclear.

In the present study, the Fabry–Perot (F-P) resonance modes and the cavity mode generated in the multilayer trench grating geometry are combined to form the hybrid modes under certain conditions. Compared with other mode-coupling approaches, the compound grating structure in this work is relatively low cost and affords easy fabrication. Moreover, based on the nature of the F-P resonance, the hybrid SPP modes always show multiple resonance orders, and all the resonant wavelengths depend linearly on the structure geometry, which offers a great design advantage for multi-band selection and regulation compared to other approaches and highlights potential to instigate plenty of applications. In the compound structure, a shallow trench was deliberately designed and fabricated to exclude interference originating from the bend loss. To evaluate the total quality of the designed geometry, various microcavity electrodynamics aspects were investigated in detail for this compound structure, specifically the absorption and radiation processes, for which the results indicate a significant reduction in loss for the entire framework. Moreover, the total absorption *Q* was calculated according to the known absorption *Q* of the F-P mode and the cavity mode based on a calculation of their weighted averages, describing the undetermined relationship between the whole and part losses, which has not been previously reported in the literature. In the process of determining the weight coefficients, the relative electric field intensity was first adopted as the standard to stipulate the effective area of the various modes. The details of the research are presented below.

## Methods

### Modeling and simulations

The compound structure was theoretically analyzed by the finite-difference time-domain method. In a series of simulations, the periodic condition was adopted as the boundary condition in both the *X*- and *Y*-directions. In addition, the perfectly matched layer condition was adopted in the *Z*-direction for the sake of extracting the reflectance spectra. The incident source is a linearly polarized plane wave, and the polarization direction is perpendicular to the gratings. The characteristic parameters for the Si material such as the permittivity and refractive index were obtained from the Palik database, and the Al parameters were obtained by CRC. Additionally, the calculations were repeatedly computed to guarantee accuracy for the simulations.

### Fabrication process

The designed multilayer trench grating was fabricated using the following process. Following surface oxidation layer chemical cleaning in hydrogen fluoride and acetone solutions, a 400-nm-thick photoresist was deposited onto a polished Si wafer by spin coating. The sample was then patterned by laser direct writing (LDW) lithography and subsequently developed in a NaOH solution for 35 s. The periodic trenches were then fabricated onto the sample by the reactive ion etching method in an SF_6_ atmosphere. The etching process proceeded for 40 s to generate shallow trenches with a depth of approximately 450 nm. The sample was then soaked in an acetone solution to clear away residual photoresist; next, the film coating process was completed in a magnetron sputtering machine, which included deposition of five alternate Al and Si films. The Si layers had a thickness of 100 nm, and the Al layers had a thickness of 50 nm, which were optimized by the simulations. The grating constant for this structure was 2.5 μm. The width ratio between the trench and grating was determined by the parameters of the LDW method. The present study generated four samples with different trench widths, specifically 1.1 μm, 1.25 μm, 1.4 μm, and 1.55 μm, respectively. For the spectrum measurement, a spectrometer equipped with a 160 mm integrating sphere was used to measure the integrated reflectance (*R*) of the fabricated samples between 500 nm and 2500 nm.

## Results and discussion

**Fig. 1:**
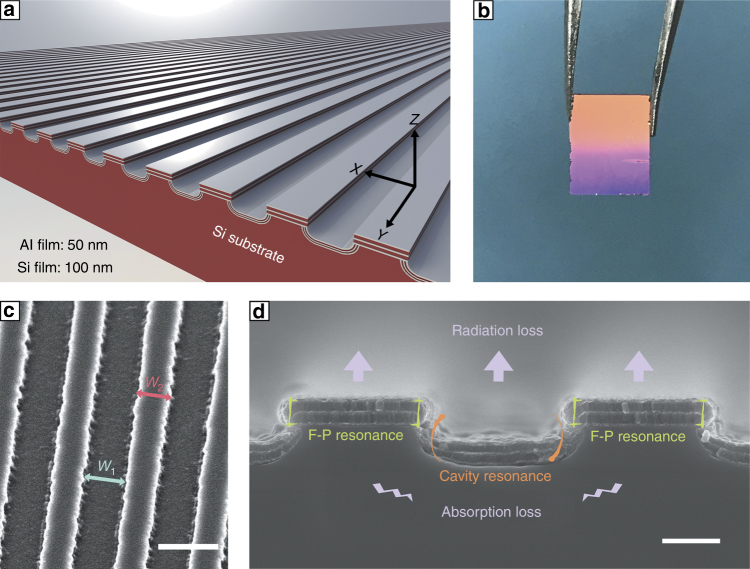
**Exhibition for sample structure. a** Diagram of the designed multilayer trench grating microstructure.**b** A photograph of the fabricated sample. **c** Top-view SEM image of the fabricated multilayer trench gratings, with a scale bar of 2 μm. **d** Cross-sectional SEM image of the fabricated multilayer trench gratings, with a scale bar of 0.5 μm. The major microcavity electrodynamics processes in this compound structure are shown

A diagram of the multilayer trench grating is shown in Fig. 1a. The compound structure is composed of two sections: a multilayer grating on the top and a multilayer trench at the bottom. Figure 1b shows a photograph of the fabricated sample. The top-view and cross-sectional scanning electron microscopy (SEM) images for the as-prepared microstructure are also shown. For convenience, the widths of the trench and grating stripe are set to *W*_1_ and *W*_2_, respectively, as shown in Fig. 1c. Figure 1d shows the local enlarged cross-sectional image of the sample and shows the grating and trench sections. However, the loss in this configuration is mainly derived from two aspects; specifically, the absorption and radiation, as shown in Fig. 1d. These properties cannot be assessed by the same method due to their different generative mechanisms. Moreover, the relationship between the losses for each individual structural part remains debatable and is carefully discussed in the following sections.

**Fig. 2:**
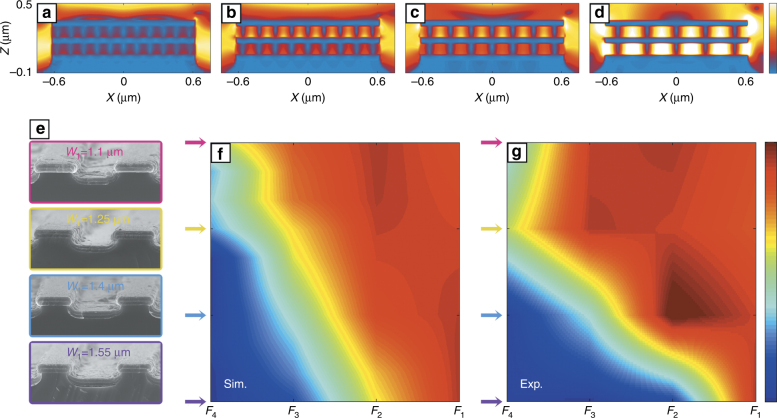
**Electric field distributions and absorption quality factors for F-P resonance modes in grating stripe. a**–**d** Electric field distributions for the multilayer grating stripe section in the *XZ* plane with F-P resonance order *m*=11, 9, 7, and 5, respectively. The trench width *W*_1_ is 1.25 μm. The color bar denotes the relative electric field intensity with a range from 0 to 2.5. **e** Cross-sectional SEM images of the fabricated multilayer trench gratings with *W*_1_=1.1 μm, 1.25 μm, 1.4 μm, and 1.55 μm, respectively. **f**, **g** Simulated and experimental $Q_{{\rm{FP}}}^{{\rm{abs}}}$ for the multilayer grating stripe section. The F-P modes from *F*_4_ to *F*_1_ are fixed as the horizontal axis, while the vertical axis represents the different *W*_1_, corresponding to Fig. 2e. The color bar denotes the values of $Q_{{\rm{FP}}}^{{\rm{abs}}}$ with a range from 1.64 to 10

The five alternate Al and Si films formed two MIM waveguides in the multilayer grating stripe to sustain a gap plasmon mode with a relatively large propagation range^24,30^. Reflection was observed at the gap terminations due to the finite length of the waveguide, thereby resulting in the formation of an inner standing wave, which is a typical F-P resonance system with two distinct features, specifically, frequency sensitivity and multiple resonance orders^34^. The order of this F-P resonance mode is simply expressed as follows^35,36^:

1 $$m = \frac{{W_{\it{2}}\beta }}{\pi } + \frac{{\varphi _r}}{{2\pi }}$$

where *φ*_*r*_ is the phase shift at the gap termination, which can be obtained by the linear fitting method, and *β* is the complex propagation constant, which can be calculated using dispersion equations derived from the symmetric waveguide theory with respect to the regular symmetric MIM waveguide, as carefully explained in our previous work^34^. Apart from the calculation method, the resonance order *m* can also be directly obtained from the simulated electric field intensity distributions. Figure 2a–d shows the various electric field distributions for the multilayer grating stripe section in the *XZ* plane with *W*_1_ = 1.25 μm. According to the standing wave distribution characteristics, the resonance orders from the short wave to the long wave were directly and successively observed as *m* = 11, 9, 7, and 5. These four modes were labeled as *F*_4_, *F*_3_, *F*_2_, and *F*_1_ and, respectively, correspond to Fig. 2a–d. The F-P modes were generated only at specific wavelengths and clearly showed frequency sensitivity for the F-P resonance. Furthermore, the resonance wavelengths were proportional to the waveguide length *W*_2_, thereby indicating that an increase in *W*_1_ resulted in resonance peak blueshift phenomenon in the spectra. Figure 2e shows the SEM images for the fabricated multilayer trench structures with *W*_1_ = 1.1 μm, 1.25 μm, 1.4 μm, and 1.55 μm, respectively. To evaluate the various losses of the compound structure, the corresponding quality factors were calculated using appropriate approaches. First, the grating stripe absorption quality factor $Q_{{\rm{FP}}}^{{\rm{abs}}}$ with negligible radiation is defined as^24^:

2 $$Q \cong \frac{\pi }{{\lambda Im(\beta )}}$$

where *λ* is the wavelength in free space; the propagation constant *β* can be solved by the dispersion equations. According to Eq. (2), both the simulated and experimental $Q_{{\mathrm{FP}}}^{{\mathrm{abs}}}$ can be calculated even if they belong to different resonance orders or different waveguide lengths. Figure 2f, g shows the results of the difference operation, which were calculated using the simulated and experimental data, respectively. The horizontal axis represents the resonance modes from *F*_4_ to *F*_1_, whereas the vertical axis represents the range of *W*_1_ from 1.1 μm to 1.55 μm. The color bar represents the value of $Q_{{\rm{FP}}}^{{\rm{abs}}}$. The series of simulations exhibited a *W*_1_ variation extent of 0.05 μm. In other words, 10 groups of simulated data were collected for the various *W*_1_ values. The $Q_{{\rm{FP}}}^{{\rm{abs}}}$ values with relatively small *W*_1_ values or low resonance orders *m* were overall larger than the others, as observed in Fig. 2f. The result shown in Fig. 2g is in good agreement with the simulated result, though less experimental data were collected compared to the simulations. Generally, a negative correlation is observed between the quality factor and the loss. A maximum $Q_{{\rm{FP}}}^{{\rm{abs}}}$ of only approximately 10 was observed, thereby implying that the absorption loss for the multilayer grating section was quite large and that the results were near the normal MIM configuration level^24^. The large absorption loss is inevitable due to the limitation of this configuration. However, with respect to the compound structure, more attention should be paid to loss evaluation for the entire framework, which can be regulated by the introduction of the trench section.

The absorption loss may be reduced by weakening the metal’s binding to the light such as in an open half-space. The energy of the multilayer trench section was confined and weakened given that its core framework embodied an asymmetric insulator–metal–insulator (IMI) structure. Furthermore, this IMI configuration can be simplified to the interface between Al and air because the light hardly penetrated the first Al film. In addition, the energy was largely concentrated in the cavity of the trench, thereby resulting in the presence of the cavity resonance mode, which is another SPP mode with multiple resonance orders^34^. However, the present study only observed the first order in the concerned waveband. Compared with the structures investigated in our previous work, the present study fabricated shallower trenches to avoid the interference originating from higher-order cavity modes. More importantly, the shallow trench can eliminate the bend loss, which will be discussed later. The corresponding electric field distributions are presented in the next section, with a propagation constant *β* which is expressed as follows:

3 $$\beta {\mathrm{ = }}k_{\rm{0}}\sqrt {\frac{{\varepsilon _m\varepsilon _{air}}}{{\varepsilon _m + \varepsilon _{air}}}}$$

where *ε*_*m*_ and *ε*_*air*_ are the permittivities of Al and air, respectively, and *k*_0_ = 2π/*λ* is the wave vector in free space. Consequently, the absorption quality factor $Q_C^{{\rm{abs}}}$ for the trench section can also be obtained by Eq. (2). For example, the simulated and experimental $Q_C^{{\rm{abs}}}$ with *W*_1_ = 1.4 μm were approximately 1105.3 and 1728.5, respectively, and were generally two or three orders of magnitude higher than that of the F-P modes. The calculated results illustrate the ability of the trench section to increase the absorption quality factor $Q_{{\rm{tot}}}^{{\rm{abs}}}$ for the entire framework. However, the detailed value remains unsolved. Additionally, the trench structure can generate additional radiation loss. As a result, it is unclear whether the total loss has increased or decreased. To solve these two problems, the total loss $Q_{{\rm{tot}}}$ for the entire structure must first be obtained.

**Fig. 3:**
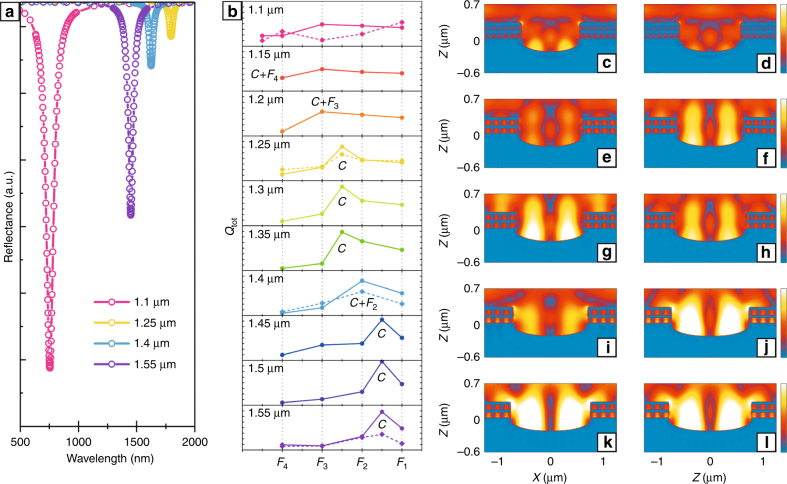
**Exhibition for SPP-mode hybridization process between F-P resonance modes and cavity modes in compound structure. a** Measured reflectance spectra for the cavity mode with *W*_1_=1.1  μm, 1.25  μm, 1.4  μm, and 1.55  μm, respectively. The spectra are successively treated by Lorentzian fitting, peak division and baseline calibration. **b** The 10 groups of simulated $Q_{{\mathrm{tot}}}$ (solid line) and 4 groups of experimental $Q_{{\mathrm{tot}}}$ (dash line) with different *W*_1_. The F-P modes from *F*_4_ to *F*_1_ are fixed as the horizontal axis. The vertical axis represents the value of $Q_{{\rm{tot}}}$; the range for each subgraph is from 0 to 80. **c**–**l** Electric field distributions for the cavity mode in the *XZ* plane with different *W*_1_, corresponding to Fig. 3b. The color bars denote the relative electric field intensity ranging from 0 to 4

Simply, the $Q_{{\rm{tot}}}$ can be extracted as *Q* = *λ*_*r*_/Δ*λ* by fitting the reflectance curve with a Lorentzian profile, where *λ*_*r*_ is the center wavelength of the resonance peak and Δ*λ* is the full width at half-maximum (FWHM) of the resonance peak in the spectrum^28,29,37,38^. Figure 3a shows the four cavity mode resonance peak spectra, which were measured for the four fabricated samples with different *W*_1_ values, and successively treated by Lorentzian fitting, peak division, and baseline calibration. The resonance wavelength of the cavity mode did not exhibit an obvious linear red- or blue-shift phenomenon following an increase in *W*_1_. However, the cavity resonance mode successively coupled with the F-P modes due to the presence of regular F-P mode blueshifts, thereby resulting in the generation of hybrid modes. Figure 3b shows the variation trend for the total quality factor $Q_{{\rm{tot}}}$, which included all the F-P modes and the cavity mode. Similar to Fig. 2f, g, 10 groups of simulated data (solid line) and 4 groups of experimental data (dash line) with various *W*_1_ are given in Fig. 3b. In addition, the F-P modes from *F*_4_ to *F*_1_ were fixed as the horizontal axis. The vertical axis represents the value of $Q_{{\rm{tot}}}$, and each subgraph ranged from 0 to 80. An increase in the trench width *W*_1_ successively merges the cavity mode *C* with the F-P modes. More concretely, when *W*_1_ = 1.1 μm, mode *C* was independent from the F-P modes; when *W*_1_ = 1.15 μm, mode *C* merged with mode *F*_4_, thereby generating hybrid mode *C*+*F*_4_. Similarly, at *W*_1_ = 1.2 μm, a hybrid mode *C*+*F*_3_ was generated. In addition, mode *C* was observed between *F*_3_ and *F*_2_ following an increase in *W*_1_ to *W*_1_ = 1.25 μm, 1.3 μm, or 1.35 μm. The formation of a hybrid mode *C*+*F*_2_ was observed when *W*_1_ = 1.4 μm. Mode *C* was observed between *F*_2_ and *F*_1_ as *W*_1_ to *W*_1_ = 1.45 μm, 1.5 μm, or 1.55 μm. These mode-coupling trends were also observed in the electric field intensity distributions. Figure 3c–l shows the electric field distributions for the cavity mode *C* in the *XZ* plane with various *W*_1_ values, which correspond to the 10 subgraphs in Fig. 3b. The relative positions between the different modes are largely represented by the resonance order *m* of the F-P modes. For instance, in Fig. 3c, the resonance order *m* is larger than 11, indicating that the cavity mode is beyond the F-P modes. Similarly, in Fig. 3d, *m* = 11, which represents the hybrid mode of *C*+*F*_4_, and so on. Therefore, the reflectance spectrum for *W*_1_ = 1.4 μm in Fig. 3a represents the peak for the hybrid mode *C*+*F*_2_. In addition to the trends for the modes coupling, more importantly, the cavity mode also has a relatively large $Q_{{\rm{tot}}}$, as well as the hybrid modes, especially in the relatively long wave range. Even compared with completely optimized Ag–GaAs–Ag structures^39^, the values of $Q_{{\rm{tot}}}$ in the present study were at least twice as large as their results under an identical thickness of the insulator layer. This is the most important conclusion with respect to the compound trench grating structure, offering a great significance for the design of hybrid plasmonic devices. The experimental results accorded well with the calculated results, though some numerical differences were observed due to deviations in the simulations and the fabrication process. These significant results illustrate the inhibitive effect on the total loss due to the introduction of the trench structure. Additionally, the concentration of energy in the cavity validated the reliability of the simplified analysis for calculation of the propagation constant *β*.

**Fig. 4:**
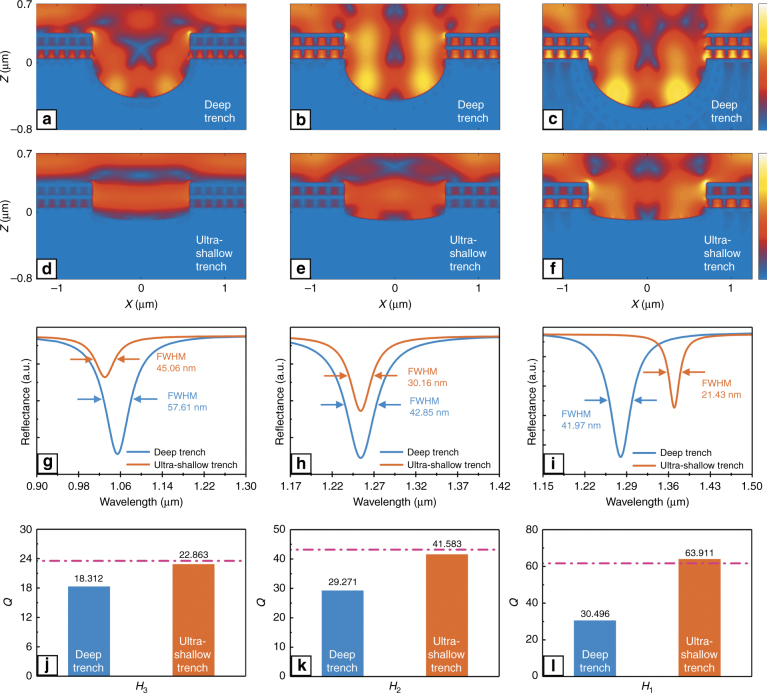
**Comparison of bend losses in various trench structures with different trench depths. a**–**c** Electric field intensity distributions for *H*_3_, *H*_2_, and *H*_1_ with the deep trenches in the *XZ* plane, respectively. The color bar ranges from 0 to 4. **d**–**f** Electric field intensity distributions for *H*_3_, *H*_2_, and *H*_1_ with the ultra-shallow trenches in the *XZ* plane, respectively. The color bar ranges from 0 to 4. **g**–**i** Comparisons between the reflectance spectra with deep and ultra-shallow trenches in *H*_3_, *H*_2_, and *H*_1_, respectively. **j**–**l** Comparisons between the *Q* factors with deep and ultra-shallow trenches in *H*_3_, *H*_2_, and *H*_1_, respectively. The pink dashed lines are the corresponding *Q* values for the normal shallow trench structure

It is remarkable that all the $Q_{{\rm{FP}}}^{{\rm{abs}}}$, $Q_C^{{\rm{abs}}}$ and $Q_{{\rm{tot}}}$ values for several hybrid modes were obtained, thereby providing a possibility for further investigation. Here, the hybrid mode *C*+*F*_4_ with *W*_1_ = 1.15 μm was renamed as *H*_3_, and the hybrid mode *C*+*F*_3_ with *W*_1_ = 1.2 μm was renamed as *H*_2_. The simulated and experimental hybrid modes *C*+*F*_2_ with *W*_1_ = 1.4 μm were renamed as *H*_1_ and $H_1^{{\rm{exp}}}$, respectively. Before discussing the microcavity electrodynamic characteristics for the compound structure, all the interference and losses must be excluded except the absorption and radiation processes. As mentioned before, the shallow trench was deliberately designed and fabricated to avoid bend loss. According to the research reported by Vlasov and McNab^40^, there is a negative exponential functional relationship between bending radius and bend loss. In the present study, the large radius represents a shallow trench. Accordingly, a series of simulations were carried out to verify whether the trench is shallow enough to safely neglect the bend loss. As shown in Fig. 4, the simulations focused on the hybrid modes *H*_3_, *H*_2_, and *H*_1_, and kept all the corresponding structure parameters the same except the trench depth. For example, Fig. 4a, d shows the electric field intensity distributions for *H*_3_ with deep and ultra-shallow trenches, respectively. The different trench shapes imply that compared with the normal shallow trench, the deep trench has an obviously smaller radius, while the ultra-shallow trench shows the opposite behavior. Figure 4g shows the reflectance spectra for *H*_3_ in the case of deep and ultra-shallow trenches, with the corresponding FWHM also given. Clearly, the latter structure shows a narrower spectrum peak compared to the former. According to the equation *Q* = *λ*_*r*_/Δ*λ*, the ultra-shallow trench structure also has a larger total *Q* factor, as shown in Fig. 4j. The pink dashed line represents the corresponding *Q* value belonging to the normal shallow trench structure, which is very close to the value obtained for the ultra-shallow structure. Since all the structure parameters are unchanged except the trench depths, the differences in the *Q* values can be completely attributed to the bend loss. The results illustrate that with increasing radius, the bend loss clearly decreases; however, if the radius is large enough, the bend loss will become very small and tend to be stable. Similarly, the hybrid modes *H*_2_ (shown in Fig. 4, second column) and *H*_1_ (shown in Fig. 4, third column) show the same conclusions. This variation trend accords well with the conclusions of Vlasov and McNab^40^, signifying that the bend loss can be safely neglected for the normal shallow trench in the present study. Based upon this condition, the absorption and radiation characteristics in the compound structure can be further discussed.

First, the total radiation quality factor $Q_{{\rm{tot}}}^{{\rm{rad}}}$ for the entire structure was calculated based on the value of $Q_{{\rm{tot}}}$ and the reflectance spectrum. According to the coupled-mode theory, the reflectance is expressed as follows^29,41^:

4 $$R(\omega ) = 1 - \frac{{A_c}}{{A_i}} + \frac{{A_c}}{{A_i}}\left| {\frac{{\frac{\omega }{{Q_{tot}^{rad}}}}}{{i(\omega - \omega _r) + \frac{\omega }{{2Q_{tot}}}}} - 1} \right|^2$$

where *ω* is the incident frequency; *ω*_*r*_ is the resonance frequency; and *A*_*c*_/*A*_*i*_ is the ratio between the effective aperture of the microstructure and the spot size of the incidence beam, which represents the light coupling efficiency^29^. The simulations followed an *A*_*c*_/*A*_*i*_ of 1. In practical measurements, the integrated reflectance is obtained by the integrating sphere to minimize energy collection waste. However, unpolarized light was used to measure the fabricated grating-shaped microstructure in the spectrometer. Consequently, half of the light was not considered in the coupling process, thereby altering the *A*_*c*_/*A*_*i*_ to 0.5 in the experiments. Subsequently, when *ω* = *ω*_*r*_, the value of $Q_{{\rm{tot}}}^{{\rm{rad}}}$ is expressed as follows:

5 $$Q_{\rm tot}^{\rm rad} = \frac{{2Q_{tot}}}{{1 + \sqrt {1 + \frac{{A_i}}{{A_c}}[R(\omega _r) - 1]} }}$$

where $Q_{{\rm{tot}}}^{{\rm{rad}}}$ is calculated by substituting the value of $Q_{{\rm{tot}}}$, *R*(*ω*_*r*_), and *A*_*i*_/*A*_*c*_ into the above equation. As mentioned above, it is still unclear whether quality factor calculations can proceed using the quality factors of individual sections for the entire framework. It is hard to treat the various types of *Q* factors by a unified approach, which is a fundamental difficulty. In terms of absorption, the compound structure in the final analysis supported two modes with different propagation constants *β*, which challenged the establishment of connections between $Q_{{\rm{FP}}}^{{\rm{abs}}}$ and $Q_C^{{\rm{abs}}}$. Clearly, the reference value for the total absorption quality factor $Q_{{\rm{tot}}}^{{\rm{abs}}}$ can first be obtained based on the following relationship^29^:

6 $$\frac{{\mathrm{1}}}{{Q_{tot}}} = \frac{1}{{Q_{\rm tot}^{\rm abs}}} + \frac{1}{{Q_{\rm tot}^{\rm rad}}}$$

**Fig. 5:**
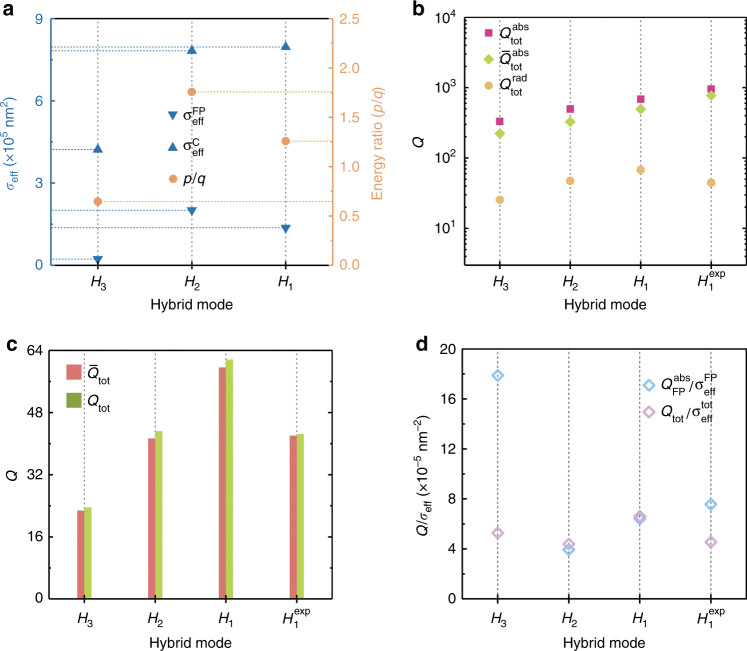
**Comparisons of various quality factors and relevant parameters in compound structure. a** Effective mode area $\sigma _{{\mathrm{eff}}}^{{\mathrm{FP}}}$, $\sigma _{{\mathrm{eff}}}^C$ (left axis) and the energy ratio p/q (right axis) in hybrid modes *H*_3_, *H*_2_, and *H*_1_, respectively.**b** Values for $\bar Q_{{\mathrm{tot}}}^{{\mathrm{abs}}}$, $Q_{{\mathrm{tot}}}^{{\mathrm{abs}}}$ and $Q_{{\mathrm{tot}}}^{{\mathrm{rad}}}$ in hybrid modes *H*_3_, *H*_2_, *H*_1_, and $H_1^{{\mathrm{exp}}}$ respectively. **c** Values for $\bar Q_{{\mathrm{tot}}}$ and $Q_{{\mathrm{tot}}}$ in hybrid modes *H*_3_, *H*_2_, *H*_1_, and $H_1^{{\mathrm{exp}}}$ respectively. **d** Comparison between $Q_{{\mathrm{tot}}}$/$\sigma _{{\mathrm{eff}}}^{{\mathrm{tot}}}$ and $Q_{{\mathrm{FP}}}^{{\mathrm{abs}}}/\sigma _{{\mathrm{eff}}}^{{\mathrm{FP}}}$ in hybrid modes *H*_3_, *H*_2_, *H*_1_, and $H_1^{{\mathrm{exp}}}$, respectively

Notably, the values of $Q_{{\rm{tot}}}^{{\rm{abs}}}$ were two orders of magnitude higher than the values of $Q_{{\rm{FP}}}^{{\rm{abs}}}$, which verified that the SPP-mode hybridization can effectively reduce the loss, whatever the total or the absorption one. Subsequently, the total absorption quality factor can also be obtained using a reasonable fitting method with the known values for $Q_{{\rm{FP}}}^{{\rm{abs}}}$ and $Q_C^{{\rm{abs}}}$. The precision of the fitting method can be evaluated based on a comparison of these two results. Therefore, the core of the problem lies in the determination of a reasonable fitting approach. For example, the total quality factor of two identical structures must be equal to the value of each independent structure, which challenges the use of the reciprocal operation such as that used in Eq. (6). As a result, the present study used the calculated weighted average as the fitting method, and the weight coefficients were determined by the energies of the two modes, which cannot be measured directly. The energy ratio between the two polarization states for the paired nano-disk structures was obtained by rotating the polarizer^28^. However, this was not applicable to our case. Accordingly, a new approach must be proposed, which must be able to avoid the difficulties of distinguishing two modes on a subwavelength scale, and precisely reflect the energy weights of the two modes in the coupling process. Due to the one-dimensional grating-shaped framework, the effective mode area *σ*_*eff*_ was defined in the *XZ* plane without considering the energy distribution along the *Y*-axis. Specifically, the use of *σ*_*eff*_ indicates that the relative electric field intensity *E* in the area, which was standardized by the incident electric field intensity, satisfies the relationship: E>*E*_max_/*e*, where *E*_max_ is the maximum electric field intensity in the mode area. This method precisely quantifies the major energy distribution area in all modes and is considered to be more reasonable than a definition based on the structure of the periodic unit cells^28^. The fitted value $\bar Q_{{\rm{tot}}}^{{\rm{abs}}}$ is defined as $\bar Q_{{\rm{tot}}}^{{\rm{abs}}}$=*p*$Q_{{\rm{FP}}}^{{\rm{abs}}}$+*q*$Q_C^{{\rm{abs}}}$, such that the weight coefficients determined by the energies can be expressed as:

7 $$p = \frac{{{\int}_{\sigma _{eff}^{FP}} {\varepsilon _d\left| {{\boldsymbol{E}}({\bf{r}})} \right|} ^2d{\bf{r}}}}{{{\int}_{\sigma _{eff}^{FP}} {\varepsilon _d\left| {{\boldsymbol{E}}({\bf{r}})} \right|} ^2d{\bf{r}} + {\int}_{\sigma _{eff}^C} {\varepsilon _{air}\left| {{\boldsymbol{E}}({\bf{r}})} \right|} ^2d{\bf{r}}}}$$

8 $$q = \frac{{{\int}_{\sigma _{eff}^C} {\varepsilon _{air}\left| {{\boldsymbol{E}}({\bf{r}})} \right|} ^2d{\bf{r}}}}{{{\int}_{\sigma _{eff}^{FP}} {\varepsilon _d\left| {{\boldsymbol{E}}({\bf{r}})} \right|} ^2d{\bf{r}} + {\int}_{\sigma _{eff}^C} {\varepsilon _{air}\left| {{\boldsymbol{E}}({\bf{r}})} \right|} ^2d{\bf{r}}}}$$

where $\sigma _{{\rm{eff}}}^{{\rm{FP}}}$ and $\sigma _{{\rm{eff}}}^C$ are the effective mode areas for the F-P mode and the cavity mode, respectively, and *ε*_*d*_ is the complex permittivity of Si. Figure 5a shows the values of $\sigma _{{\mathrm{eff}}}^{{\mathrm{FP}}}$ and $\sigma _{{\rm{eff}}}^C$ for the three hybrid modes, specifically *H*_3_, *H*_2_, and *H*_1_ (left axis), and the energy ratio *p*_1_/*q*_1_ of the hybrid modes (right axis). Mode $H_1^{{\rm{exp}}}$ can invoke the coefficients of mode *H*_1_ in the fitting process. Accordingly, the value of $\bar Q_{{\rm{tot}}}^{{\rm{abs}}}$ can be fitted by calculating the weighted average using the above coefficients. Figure 5b shows the values of $Q_{{\rm{tot}}}^{{\rm{rad}}}$, $Q_{{\rm{tot}}}^{{\rm{abs}}}$, and $\bar Q_{{\rm{tot}}}^{{\rm{abs}}}$ for several hybrid modes. Considering the latter two are one order of magnitude higher than the other quality factors and their deviations easily increase, the results for the fitted $\bar Q_{{\rm{tot}}}^{{\rm{abs}}}$ are in very good accordance with the values for $Q_{{\rm{tot}}}^{{\rm{abs}}}$. Subsequently, the fitted $\bar Q_{{\rm{tot}}}^{{\rm{abs}}}$ was adopted to solve the total quality factor using Eq. (6), which is represented as $\bar Q_{{\rm{tot}}}$. Figure 5c shows comparisons between the values of $\bar Q_{{\rm{tot}}}$ obtained by the fitting method and the results directly extracted from $Q_{{\rm{tot}}}$=*λ*_*r*_/Δ*λ*. In general, the results for $\bar Q_{{\rm{tot}}}$ were close to the values for $Q_{{\rm{tot}}}$, which indicates the reliability of the presented approach to calculate the weighted average and the accuracy of the weight coefficients in the fitting process. By virtue of such a pure mathematical method, one can estimate for the first time the quality factor for the entire framework from the known quality factors for the individual sections, which offers a new insight for investigating various kinds of plasmonic modes, even with different propagation constants.

Additionally, the trade-off between loss and energy confinement is one of the most important principles and considerations when designing a high-performance plasmonic device^30^. Referring to the concept of the Purcell factor^42,43^, the values of $Q_{{\rm{tot}}}$/$\sigma _{{\mathrm{eff}}}^{{\rm{tot}}}$ ($\sigma _{{\mathrm{eff}}}^{{\rm{tot}}}$=$\sigma _{{\mathrm{eff}}}^{{\rm{FP}}}$+$\sigma _{{\mathrm{eff}}}^C$) were characterized to evaluate the relationship between energy localization and loss for the entire framework, as shown in Fig. 5d. Similar to previous calculations, the $H_1^{{\mathrm{exp}}}$ mode invokes the relevant parameters of the *H*_1_ mode to obtain the ratio. Moreover, the values for $Q_{{\rm{FP}}}^{{\rm{abs}}}/\sigma _{{\mathrm{eff}}}^{{\rm{FP}}}$ were also characterized for reference. As known, the MIM has a great ability to confine light, thereby generating a relatively small $\sigma _{{\mathrm{eff}}}^{{\rm{FP}}}$. In Fig. 5d, the two groups of data showed the same order of magnitude, which illustrates the strong energy confinement and relatively low loss in the compound multilayer trench grating structure.

## Conclusions

In summary, a MIM configuration strongly confines light into a relatively small space despite the generation of a relatively large absorption loss. In contrast, a semi-open asymmetric IMI configuration generally exhibits serious radiation loss instead of absorption. In this work, a compound multilayer trench grating structure was demonstrated, which includes grating stripes and shallow trench sections. The former geometry formed F-P resonance modes in the MIM waveguides with frequency sensitivity and multiple resonance orders. Serious absorption loss was evaluated by calculating the relevant quality factor. However, based on the cavity mode generated in the trenches, the latter geometry showed a relatively low absorption loss. The shallow trench in the present study was deliberately designed and fabricated to avoid bend loss. In addition, the total loss for the entire structure decreased following the introduction of the trench configuration, thereby illustrating the ability of the compound framework to simultaneously consider absorption and radiation loss. Furthermore, according to investigations of the hybrid modes, which were formed by the F-P and cavity modes, the total quality factor was fitted by calculating the weighted average of the partial values of the individual sections, which described their previously undetermined relationship. In this process, the effective area of the different modes was stipulated by the relative electric field intensity, which was more reasonable than the common definition based on a unit structure. Finally, the ratio between the quality factor and the effective mode area for the compound framework was characterized. The results indicate the ability of the multilayer trench grating to reduce loss without weakening energy confinement, which can fill a gap in the field of hybrid SPP-based high-quality plasmonic structures.
